# Etiology and Treatment of Pneumonia

**Published:** 1875-04

**Authors:** 


Etiology and Treatment of Pneumonia.—* * * The discussion was commenced by Dr. Austin Flint:

Pneumonia is a disease showing marked diversities in its nature, in different places, and at different times. To-day we hesitate to accept anything that cannot be proved to a demonstration, but our progenitors realized that at different times a disease might show marked changes in its characteristics. Malaria, in some places, is of a very fatal type, and scarlet fever is a disease that differs greatly with the epidemic, not only in its severity, but in its tendency to complications. Pneumonia in the Southern States is of a graver nature than it is in the North, and at the present time in this city it is more grave than we usually consider it. Several cases have come under my own observation during the month of January, in which death has taken place in a few days. Before referring to the treatment, I shall read notes of a case that I treated in 1837. The man was in good health previous to the attack, and about twenty-two years of age. I bled him to the extent of thirty ounces, and then gave him five grains of calomel every four hours. In the evening the pain in the side was not so severe, but still existed. He was again bled, nine ounces being taken. On the following day the pain was relieved. In six days from the attack he was convalescing, and in sixteen days recovery was complete. Louis reported a number of cases of pneumonia in which he used blood-letting. He did not consider it necessary to consider its effect on the mortality, taking for granted that there could be no doubt as regards its beneficial effect in that way, but what he wanted more particularly to determine was, whether the bleeding did or did not shorten the disease. His conclusions were that bleeding not only had a happy effect, but it shortened the disease by four or five days. It never arrested the disease at once, however. He found that if the blood was taken from the patient late in the disease, it had an adverse influence, and tended to lengthen it. Jackson, of Boston, was of the opinion that if the patient were bled on the first day of the disease, its duration would be shortened from fourteen to eleven days. Louis reported seventy-eight cases, and had a mortality of twenty-eight deaths. He afterwards reported twenty-nine other cases with four deaths. Jackson reported fifty-one case with eight deaths. His treatment was the same as that of Louis.

When pneumonia is limited to a single lobe, it is important to bear in mind that the cases prove fatal only by complication and extension of the inflammation, and it is for us to consider whether bleeding does or does not tend to avert these complications. Lately we have had the scheme presented to us of lowering the heat of the patient by the use of external cold. Internally, quinia, in five-grain doses, three times a day, I have found to be of decided benefit. Death occurs nearly always from asthenia, and the use of alcoholics, in the latter stages, I think is undoubtedly indicated.

Attention has been recently called to a cause of pneumonia that we do not ordinarily take into account. I mean that arising from the effects of sewer gas. Near London they deemed it necessary to open a ventilator for a sewer in close proximity to a high school; the principal most emphatically protested, but without effect. Shortly after a number of cases of pneumonia developed, and it was found necessary to close the school. Previous to this the school was perfectly healthy, and after the removal of the sewer ventilator no further sickness occurred. We are thus led to the inference that the sewer gas was the direct cause in this case.

Dr. W. H. Thompson read the histories of five cases of pneumonia that came under his observation at Bellevue Hospital. They were all treated on the antipyretic plan, bags containing ice being applied to the chest, to reduce the temperature of the patient. In all the cases there was a decided reduction of temperature, and with the intermission of the use of the ice the temperature again rose. Of the five cases reported only one proved fatal. He gave ten grains of carbonate of ammonia every two hours, and one grain of quinia every hour.

Dr. A. L. Loomis said: The two important things in the consideration of pneumonia are, the etiology and treatment. Of all the causes, age ranks first; we see it usually between twenty and forty, and after sixty years of age. I mean croupous pneumonia. The pneumonia of children is broncho-pneumonia. Climate also exercises an influence, and particularly if it is liable to sudden changes. Here we do not see much of idiopathic pneumonia. It is true we find it in the books, but so rare do I find it that I am tempted to question its existence. The great number of cases occur in those who are not in full health. In this city we are all staggering under the influence of malaria, and a slight exposure lights up the disease. Anything that debilitates the system has a similar effect, such as sewer gas, septicæmia, pyæmia, rheumatism, etc. There are few who would care to bleed, when pneumonia is due to debility, and as I said before, it is with that form that we have most to deal with. The use of ice-bags I am decidedly opposed to. I have not had as large an experience as I should wish, but so far my experience is against it; for though the temperature may be reduced, I find that there is a tendency to an extension of the inflammation.

I found recently, on coming on duty at Mount Sinai Hospital, several cases treated in this way, and they all died. The cause of heat is the metamorphosis of tissue, and the drug, in my experience, that controls this metamorphosis is the sulphate of quinia. I give it in ten-grain doses. When the heart begins to flag, and we have resulting œdema of the lungs, and other signs of debility, I rely on alcohol freely given to tide the patient over. I find it more reliable to stimulate the heart than any other drug I have ever used.—*Medical and Surgical Reporter, March.*

